# PES/POSS Soluble Veils as Advanced Modifiers for Multifunctional Fiber Reinforced Composites

**DOI:** 10.3390/polym9070281

**Published:** 2017-07-13

**Authors:** Gianluca Cicala, Ignazio Blanco, Alberta Latteri, Giulia Ognibene, Francesco Agatino Bottino, Maria Elena Fragalà

**Affiliations:** 1 Department of Civil Engineering and Architecture and INSTM UdR, University of Catania, V.le A. Doria 6, 95125 Catania, Italy; iblanco@dii.unict.it (I.B.); alatteri@unict.it (A.L.); 2 Department of Chemical Science and INSTM UdR, University of Catania, V.le A. Doria 6, 95125 Catania, Italy; giuliaognibene@live.com (G.O.); fbottino@dii.unict.it (F.A.B.); me.fragala@unict.it (M.E.F.)

**Keywords:** electrospinning, epoxy, POSS, composites

## Abstract

Novel polyhedral oligomeric silsesquioxanes (POSS)-filled thermoplastic electrospun veils were used to tailor the properties of the interlaminar region of epoxy-based composites. The veils were designed to be soluble upon curing in the epoxy matrix, so that POSS could be released within the interlaminar region. Three different POSS contents, varying from 1 to 10 wt %, were tested while the percentage of coPolyethersulphone (coPES) dissolved in the epoxy resin was kept to a fixed value of 10 wt %. Good quality veils could be obtained at up to 10 wt % of POSS addition, with the nanofibers’ diameters varying from 861 nm for the coPES to 428 nm upon POSS addition. The feasibility of the soluble veils to disperse POSS in the interlaminar region was proved, and the effect of POSS on phase morphology and viscoelastic properties studied. POSS was demonstrated to significantly affect the morphology and viscoelastic properties of epoxy composites, especially for the percentages 1% and 5%, which enabled the composites to avoid POSS segregates occurring. A dynamic mechanical analysis showed a significant improvement to the storage modulus, and a shift of more than 30 °C due to the POSS cages hindering the motion of the molecular chains and network junctions.

## 1. Introduction

Thermosets are widely used in polymer matrix-reinforced composites because of their easy processing and good mechanical properties. However, the inherent brittleness of thermosets led to the development of different toughening strategies. Among the most recent toughening approaches, the use of electrospun veils is particularly advantageous.

Some authors have focused on non-soluble electrospun fibers based on the use of Nylon fibers. Akangah et al. [1] showed that nylon-6,6 veils improved the impact force by about 60%, while reducing the damage growth rate to one-half. The nylon-6,6 veils also reduced the impact damage growth rate with impact force. Palazzeti et al. [2] confirmed similar results. Beckermann et al. [3] reported a detailed study on nylon-based commercial veils, which are based on the same concept.

Other authors have proposed a different approach, based on the use of soluble veils that dissolve under resin curing and phase separately, generating specific phase morphology. Li et al. [4] showed that polysulfone veils can be dissolved in the interlaminar region, giving rise to a particulate morphology that led to toughness improvements of up to 42%. Cicala et al. [5] showed that by varying the veil content and the resin formulation, a full range of morphologies in the interlaminar region can be obtained.

Recently, electrospun fibers that can be used to selectively disperse carbon nanotubes in the interlaminar region upon veil dissolution were reported [6]. Cicala et al. [7] extended this concept in order to develop a multifunctional composite. Lionetto et al. [8] showed that this approach can be used to orient carbon nanotubes in a thermosetting matrix. Hamer et al. [9] showed that nylon electrospun fibers filled with carbon nanotubes performed better in improving toughness compared to unfilled nylon electrospun fibers. Despite the interest for this approach, to the best of our knowledge, only carbon nanotubes have been studied.

Nanofillers have shown their efficiencies for the toughening of epoxy resin, too. Gojny et al. [10] studied the effect of carbon nanotubes on the fracture resistance of epoxy resins. They showed an increase of toughness from 0.65 MPa·m^1/2^ for the unmodified resin to 0.80 MPa·m^1/2^ by adding 1% of multiwall carbon nanotubes functionalized with amine groups. Chul Kim et al. [11] reported a similar improvement of fracture resistance by adding carbon black or nanoclay. Commercial core-shell rubber nanoparticles were reported as efficient toughening modifiers by Carolan et al. [12]. In situ-formed silica nanoparticles were shown to improve both toughness and the interfacial bonding between carbon fibers and matrix [13].

Among the different nanofillers that can be added to epoxy resins for tailoring their properties, polyhedral oligomeric silsesquioxanes (POSSs) are of high interest. POSSs are of interest as they can combine a hybrid inorganic–organic composition with nanosized silica-like cage structures. In addition to that, their dimensions are comparable to those of most polymeric segment or coil, and they can be bound to polymer chains. These properties allow POSS to reinforce systems at the molecular level. Finally, as for other nanoparticles, the large surface area allows the use of only small amounts to cause significant changes in the properties of the matrix [14]. In several reviews, the advantages of POSS as functional fillers were discussed. POSS can be used to improve: glass transition temperature (*T*_g_) [15,16], fire resistance [17], resistance to photo-degradation [18], and electrical and mechanical properties. POSS is truly a multifunctional filler. Recently, Konnola et al. [19] reported about the efficiency of POSS for the tailoring of complex epoxy/rubber blends.

The effect of POSS on the viscoelastic properties of an epoxy network is discussed in the literature. Lee and Lichtenhan [15] observed an increasing and broadening of the glass transition, which were explained as the result of the nanoscopic size of these POSS cages and their ability to hinder the motion of the molecular chains and network junctions. Many other authors reported POSS to lead to *T*_g_ increases for epoxy resins [20]. However, a reduction of *T*_g_ with increasing POSS content is also reported [21]. Frank et al. [22] investigated the influence of the multiscale (i.e., nano and micro) dispersion of POSS, hypothesizing that well-dispersed POSS tended to plasticize polymers, lowering *T*_g_ by increasing free volume and reducing interaction between polymer chains. This result contrasted with other findings, but it must be observed that the amino-propylisobutyl POSS used by Frank et al. had a long aliphatic chain, which might have affected the result.

Some authors have reported the addition of POSS to electrospun fibers [23], but to the best of our knowledge, no report on the electrospinning of POSS dispersed in polyethersulphone has ever been reported. In addition to that, no one has ever tried to selectively disperse POSS by using soluble fibers or veils. The aim of the present paper is to prepare electrospun coPolyethersulphone (coPES) veils embedding POSS fillers, and to verify the use of these veils to selectively disperse POSS in the interlaminar region of composites manufactured by resin infusion.

## 2. Experimental

Here, we report for the first time the use of POSS-filled electrospun soluble veils for the delivery of POSS into the interlaminar region of advanced composites manufactured by resin infusion. The proof of the concept is achieved combining both miscroscopic and spectroscopic techniques. In addition to that, the effect of POSS on the viscoelastic properties of the composites are discussed.

### 2.1. Materials and Methods

#### 2.1.1. Materials

The epoxy resin used was diglycidyl ether of bisphenol A (DGEBA) supplied by Shell Chemical, Akron, OH, USA. The curing agent was 4,4′-methylene bis(2,6-diethylaniline) (MDEA) supplied by Lonza, Basel, Switzerland. The thermoplastic polymer was a coPolyethersulfone, synthetized by the authors, with an average molar mass of 9000 g/mol (coPES9k). The details regarding the synthesis and characterization of coPESs are discussed elsewhere [24,25,26]. Plain carbon fabrics (C-200 T from Prochima, Italy) with an areal weight of 200 gsm were used for the preparation of the reinforced samples. The unmodified epoxy matrix was prepared by mixing, in stoichiometric amounts, the hardener and the epoxy monomer at 80 °C for 30 min.

#### 2.1.2. POSS Synthesis

4-chlorophenyl hepta isobutyl–POSS was synthesized by the Corner Capping Reaction of isobutyltrisilanol with the suitable 4-chlorophenyltrimethoxysilane, which was prepared from the appropriate Grignard reagent and Si(OCH_3_)_4_ [27,28]. The isobutyltrisilanol (iC_4_H_9_)_7_–Si_7_O_9_(OH)_3_ was prepared according to methods described in the literature [29]. Tetrahydrofuran (THF) was distilled over a Na–benzophenone mixture.

**Figure 1:**
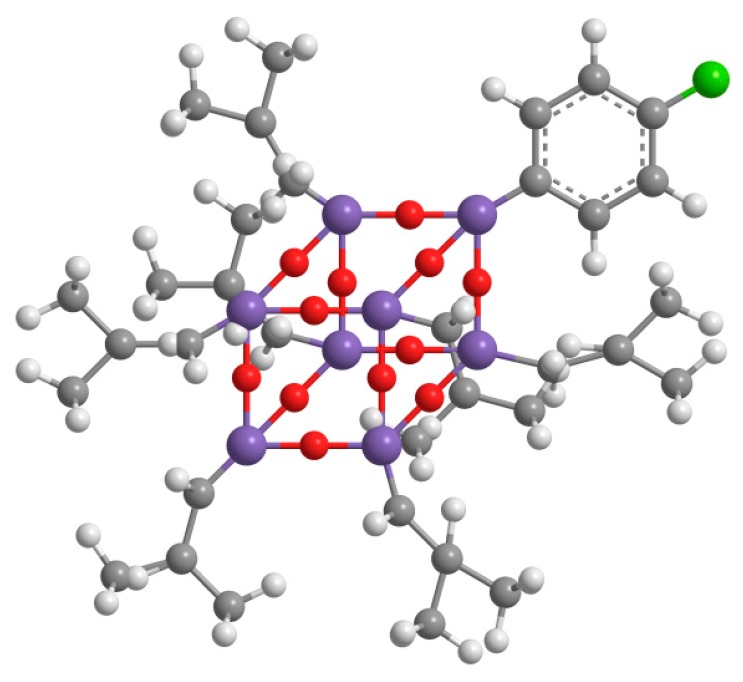
The chemical structure of the 4-chlorophenyl hepta isobutyl–polyhedral oligomeric silsesquioxane (POSS) synthetized.

Isobutyltrisilanol (3.95 g, 5.0 mmol) was dissolved in 50 mL of ethanol and 4-chlorophenyltrimethoxysilane (1.21 g, 5.2 mmol) and 2.0 mL of tetramethylammonium hydroxide were added under stirring. The solution was mixed by stirring at room temperature for 24 h. The resulting solid was then filtered and washed with 10 mL of anhydrous ethanol. Drying under reduced pressure was then performed, and the white solid obtained was crystallized from a THF/MeCN mixture to obtain 3.60 g of the desired compound (77.7% yield). The chemical structure of the synthetized POSS is reported in Figure 1.

#### 2.1.3. Electrospun Veils Preparation

The coPolyethersulphone (coPES) veils were prepared by dissolving the coPES (5.00 g) powder in a solvent mixture (5.00 mL *N*,*N*-dimethylformamide (DMF) and 5.00 mL of toluene). The solution obtained was mixed for 2 h at 40 °C. This solution was positioned in a 3 mL medical syringe. The processing parameters to electrospin the coPES veils were as follows: flow rate of 60 µL/min, voltage of 21 kV ddp, and a needle–collector gap of 10 cm. The collector used was a rotating drum (200 rpm) covered with antistatic paper. Electrospinning was carried out on an EC-DIG electrospinning apparatus purchased from IME Technologies, Geldrop, The Netherlands.

For the preparation of the coPES fibers with POSS embedded in them, three different solutions (with 1%, 5%, and 10% of POSS to PES, respectively) were prepared. The starting solution, for all of the three cases, was that one of coPES prepared by using the same procedure described above. When the polymer was completely solubilized, POSS powder (in the three different percentages) was added into the solution and uniformly dispersed by using the ultrasaunder UP 200 Ht by Hielscher, Teltow, Germany. After 1 h, the three solutions were electrospun with the same conditions used for coPES.

#### 2.1.4. Composites Manufacturing

Six layers of dry carbon fabrics with electrospun veils were laid down on a flat steel plate. The proper sealing was ensured by placing an adhesive silicone tape around the perimeter of the layered stack. A flexible vacuum bag was placed on top of the assembly. An inlet tube and an outlet tube were placed inside the vacuum bag. The vacuum was applied, through a valve, while the inlet valve was closed to compact the layers and to remove excess air. After the proper fabric degassing was ensured, the stacked layers were placed in an oven preheated to 130 °C. The epoxy resin was then vacuum infused into the stacked layers. The temperature was kept at 130 °C for 30 min, and then increased by 2 °C/min up to 180 °C and held at that value for 3 h. Considering the weight of the carbon fabric (i.e., 200 gsm), the correct amount of coPES veil was laid in the interlaminar regions to obtain 10 wt % of the toughener in the final laminate.

### 2.2. Characterization

#### 2.2.1. Hot Stage Microscopy Analysis of the Veils’ Dissolution into the Unmodified Epoxy Matrix

A hot-stage microscopy analysis was carried out to monitor the dissolution of coPES veils in the epoxy resin formulation at different temperatures. The Linkam THMS 600 hot-stage with a TP-90 controller (Tadworth, UK) was fitted to an Olympus BX60 optical microscope (Milan, Italy). The unmodified epoxy resin (i.e., an uncured mixture of DGEBA and MDEA) was preheated to the testing temperature. After that, a single drop of resin was laid down on a thin glass microscope slide. The coPES veil was then placed on top of the resin droplet, after which another thin glass slide was placed on top of the veil wetted by the resin droplet. The specimen was held in the hot-stage at the testing temperature for the time needed to observe the complete dissolution of the veil. This procedure was carried out for testing temperatures of 80, 90, 100, 110, 120, and 130 °C.

#### 2.2.2. Dynamic Mechanical Analysis (DMA) of Cured Composites

The viscoelastic behavior of the materials was investigated using a DMTA device (TRITEC by Triton Technology, Lincolnshire, UK) by single cantilever geometry and using samples of size (20 × 10 × 3) mm^3^. The tests were carried out at 1 Hz with a 2 °C/min heating rate ranging from −100 to 130 °C, using liquid nitrogen for sub-ambient scans.

#### 2.2.3. Scanning Electron Microscopy (SEM)

Micrographs were obtained by using a SEM: Zeiss EVO, Cambridge (UK). SEM analysis was carried out on the electrospun veils and on the cured laminates. The electrospun veils were gold sputtered before the analysis without any other pre-treatment. The diameter of the nanofibers was determined from the SEM images with the software Fibraquant. The interlaminar regions of the composite laminates were examined by SEM after polishing before etching. In one case, a crio-fractured surface was also analyzed for comparison purposes. An acid mixture was used to etch the epoxy phase. The effect of the etching procedure was to increase the contrast between the thermoplastic and epoxy phases. The etching treatment was carried out by the immersion of the samples in the acid mixture, followed by a stirring time of 20 min. The samples were always washed with water after etching, and were then sputtered with gold. The etching time did not alter the phase morphology [5].

#### 2.2.4. Thermogravimetric Analysis (TGA)

The thermal degradations of the studied composites were carried out in a Thermogravimetric Analyzer TGA 1 Star System (Mettler, Greifensee, Swizterland). Samples of about 5 × 10^−3^ g, put in open alumina crucibles, were degraded in dynamic heating conditions (10 °C·min^−1^) in the temperature range of 25–800 °C in flowing nitrogen (0.02 L·min^−1^), and their weights were measured as a function of temperature. The error in the mass determination due to the reduction of the buoyancy force on increasing temperature [30] was corrected by a thermogravimetric (TG) run with an empty pan (blank), which was preliminarily performed in the same experimental conditions used for all of the samples. The blank curve obtained was therefore subtracted from those of the samples. This procedure allowed us to obtain the corrected degradation TG curves. At the end of each experiment, these data were used to plot the percentage of undegraded sample, (1 − *D*)%, as a function of temperature, where *D* = (*W*_o_ − *W*)/*W*_o_, and *W*_o_ and *W* are the weights at the starting point and during scanning, respectively.

## 3. Results and Discussion

### 3.1. Effect of POSS Addition on Veil Quality

**Figure 2:**
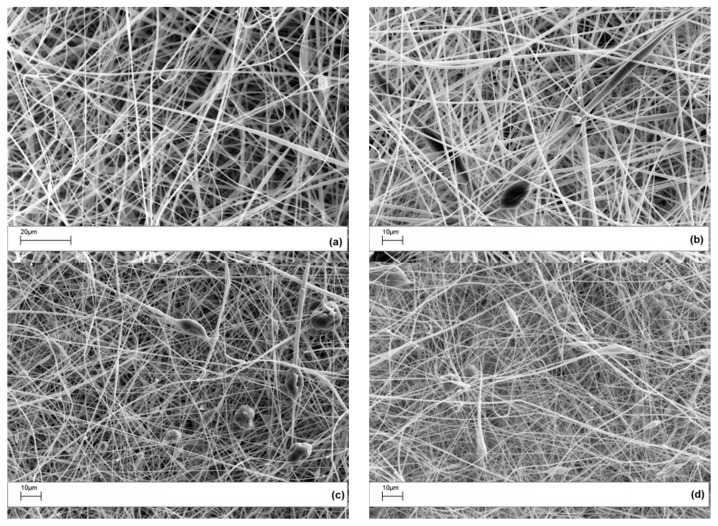
SEM images of electrospun PES veils: (**a**) coPES; (**b**) coPES/1% POSS; (**c**) coPES/5% POSS; and (**d**) coPES/10% POSS.

The morphology of the electrospun veils with different POSS contents is shown, at low magnification, in Figure 2.

**Figure 3:**
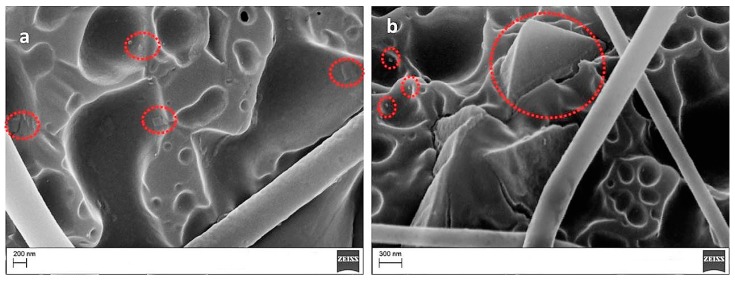
SEM images of electrospun PES containing 5% (**a**) and 10% POSS (**b**) (POSS micro and nanocrystallites are indicated by red dotted circles).

For the 0 wt % and the 1 wt % POSS veils, the electrospun fibers appear uniform and with very few beads. When the POSS content increased to 5 and 10 wt %, more beads in the micrometer range appeared. By increasing the POSS loading in the PES, large agglomerates tend to crystallize by themselves, since a uniform dispersion of POSS is achieved by limiting the relative percentage [31]. In fact, at higher concentrations, POSS units in polymers tend to aggregate or even form crystallites. In Figure 3, high magnification SEM images revealed that beads found in the PES veils with a POSS content of 5 and 10 wt % (Figure 2c,d) had a porous structure with squared POSS nanocrystallites (Figure 3a, 5% POSS) and microcrystallites (Figure 3b, 10% POSS) visible within the pores.

An EDX analysis confirmed a high Si content in the composite nanofibers upon increasing the POSS loading from 1 to 10 wt % (Table 1).

It is noteworthy that the chlorine presence in the POSS structure is detectable, by EDX, in the sample containing the high percentage of POSS.

**Figure 4:**
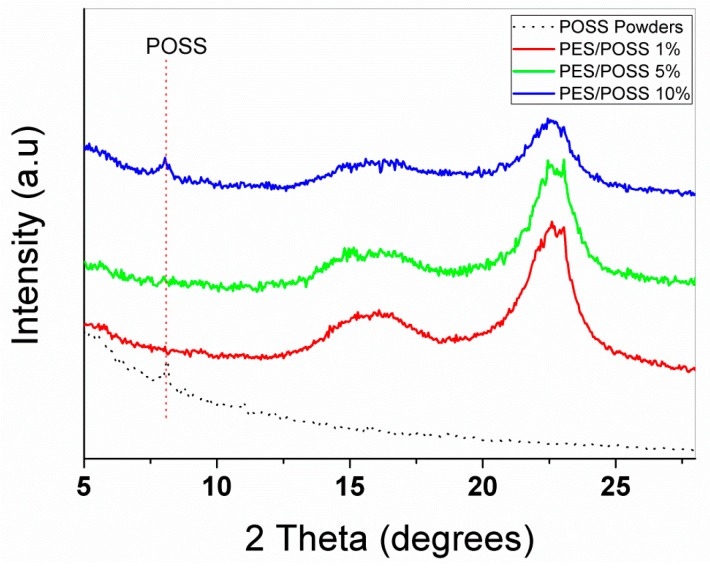
XRD patterns of electrospun PES containing variable wt % of POSS (solid line). The POSS powders’ diffractogram is shown for comparison (dotted line).

More evidence of the presence of crystallised agglomerates of POSS in the PES veils is provided by the XRD analysis. The diffraction spectrum reported in Figure 4 shows a peak at 2Q = 8° consistent with the presence of POSS cages in the materials (corresponding to the lattice spacing of 10.8 A), thus confirming that POSS segregates in the hybrid composites.

**Figure 5:**
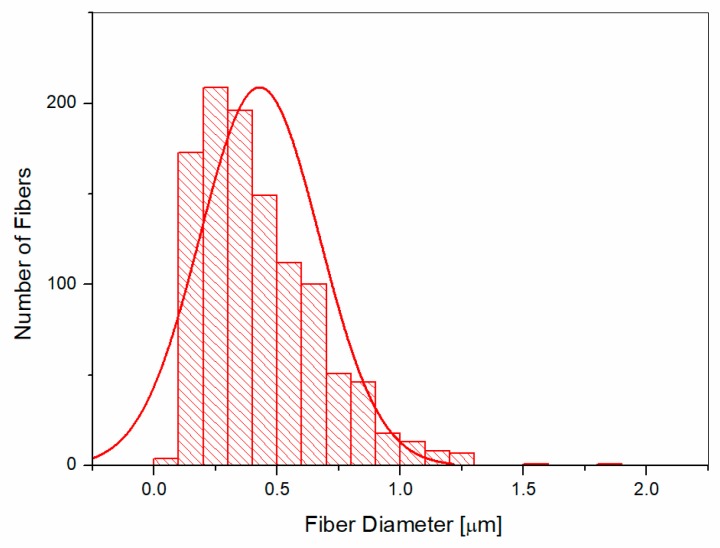
Histogram and Gaussian distribution of the fiber diameters for the veil with 10 wt % POSS when including the beads in the calculation.

**Figure 6:**
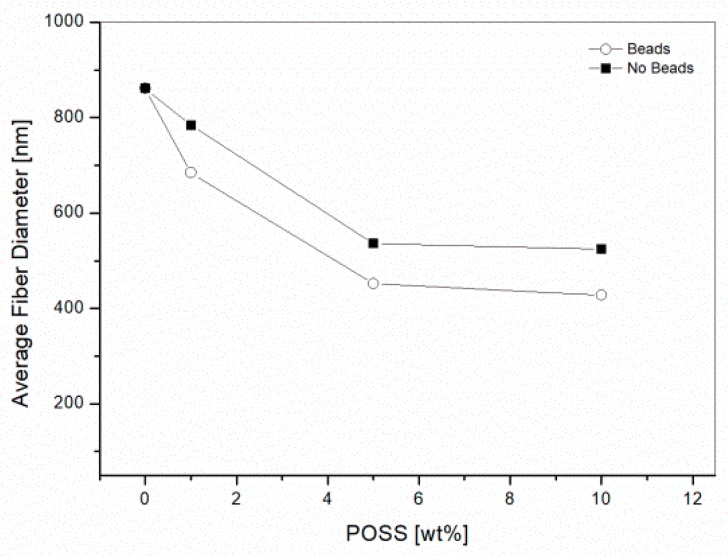
Average fiber diameter for the veil with 10 wt % POSS when excluding the beads from the calculation.

The average fiber diameters for the electrospun veils were calculated by the SEM images with the image software analysis Fibraquant, which has been specifically developed for the analysis of electrospun fibers. The software allows for the exclusion of the beads from the calculation. An example of the distribution graph for the average fiber diameters in the case of the coPES veils is shown in Figure 5. The results for the veils, excluding the beads’ contribution, is reported in Figure 6. The average fiber diameters varied from 861 nm for the coPES to 428 nm for the veil with a 10 wt % of POSS, confirming that upon the POSS’ addition the nanofibers get thinner, but also more beads appear. When the beads are considered in the measurement, the trend does not change, but a smaller apparent average fiber diameter is calculated.

The decrease of the coPES/POSS nanofiber diameters could be explained by considering that the silsesquioxane molecules, containing Si atoms, increased the conductivity of the solutions. Increasing the solution’s conductivity is known to result in thinner fibers, because the increased charge carrying capacity of the jet results in higher tension on the jet with the applied electric field [32]. Similar results were reported by Cozza et al. [23] for a cellulose acetate nanofiber loaded with epoxycyclohexylisobutyl POSS.

### 3.2. Veil Dissolution in Neat Epoxy and Morphological Analysis of the Cured Composites

**Figure 7:**
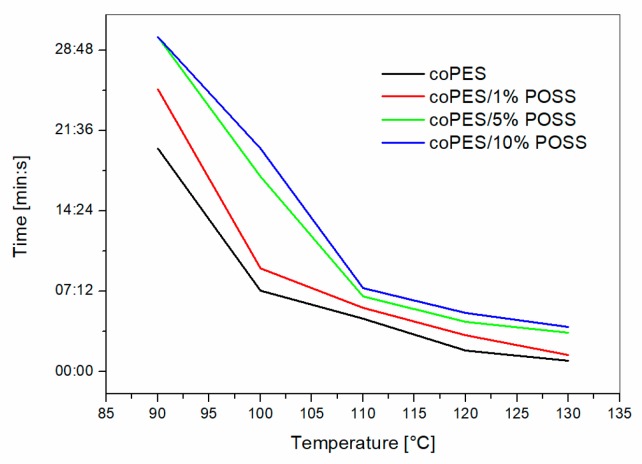
Time to dissolution for different POSS contents as observed by the hot-stage analysis.

Hot-stage microscopy was used to evaluate the dissolution rate in the uncured epoxy matrix of the electrospun veils at different temperatures. This is a first test used to assess the behavior upon the curing of the soluble veils. The time required to obtain the full dissolution of the veils is summarized in Figure 7.

The graph shows that increasing the POSS content resulted in a delayed time for the veil’s full dissolution. Similar results were found previously for higher molar mass coPES [5]. The epoxy resin acts as a solvent for the coPES-based veil. Veil dissolution follows the rules of polymer dissolution into a solvent. Therefore, the addition of a filler that might interact with the dynamics of polymer chains, hindering their motions, can result in a slower diffusion rate, and thus in a lower dissolution rate. This is the case for the nanoscopic POSS cages, which can interact with polymer chain diffusion. It is important to note that the system with 1% POSS showed minor differences with the coPES only when tested at high temperature (i.e., 130 °C), while the other two formulations had a higher dissolution time even at this temperature.

**Figure 8:**
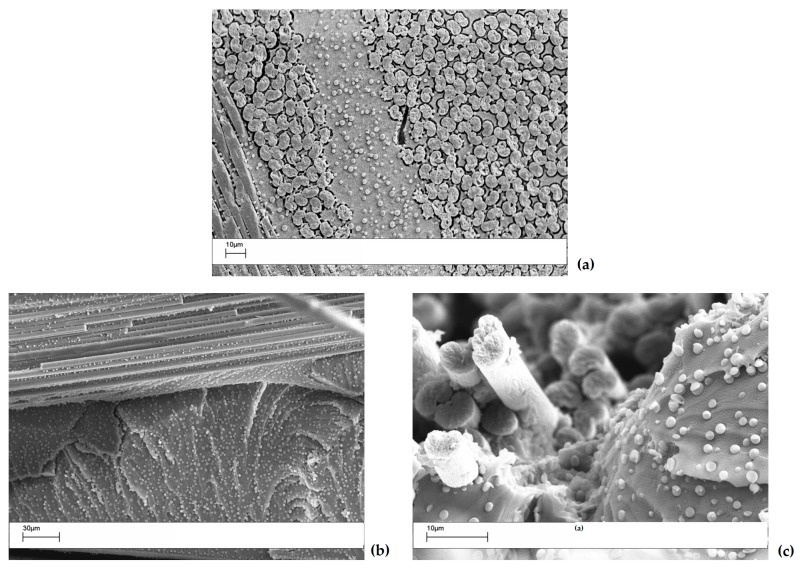
SEM analyses of the interlaminar region for the composite with a 10 wt % coPES veil dissolved in it: (**a**) polished section of the laminates; (**b**,**c**) crio-fractured surfaces for the same laminate.

The interlaminar region of the cured laminate was analyzed by SEM to monitor the dissolution and phase separation of the coPES nanofibers. Figure 8 shows the interlaminar region for the polished and crio-fractured sample with a 10 wt % of coPES veil after the full cure cycle. The sample showed the full dissolution of the nanofibers with the development of a particulate morphology, with the coPES-rich particles having an average diameter of 1–3 µm. The coPES-rich particles are found in the intralaminar region too, confirming the high level of polymer diffusion upon its dissolution during the curing cycle, which lead to the phase separation occurring between the carbon fibers’ tow.

**Figure 9:**
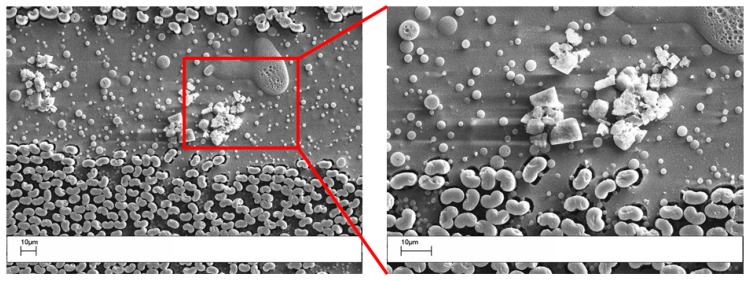
SEM of the interlaminar region for the composites with dissolved in 10 wt % of coPES/1% POSS. On the right is the magnification (1000×) view of a POSS micro agglomerate.

The interlaminar region of the sample with 10 wt % of coPES/1% POSS showed distinctive features with respect to that with a coPES veil (Figure 9). In this sample, a bimodal distribution of coPES rich particles appeared, with small 1–3 µm particles and bigger 5–7 µm particles (for magnification 500× and 4000×). In addition to that, regions with phase-inverted morphology and squared micro POSS agglomerates appeared.

**Figure 10:**
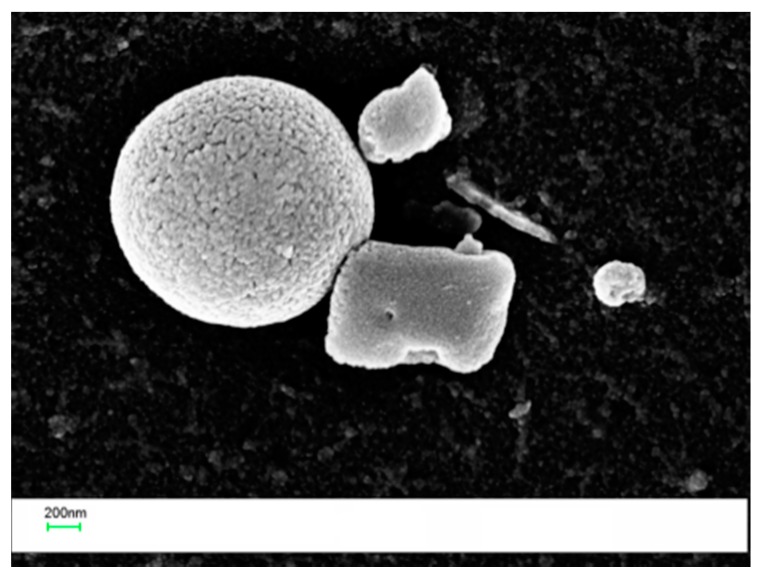
High resolution SEM images of the laminate obtained with the coPES/1% POSS veil.

A higher resolution SEM coupled with EDX analysis was performed on the same sample. The analysis at a magnification of 80,000× revealed the presence of nanoscaled POSS crystallites (Figure 10).

**Figure 11:**
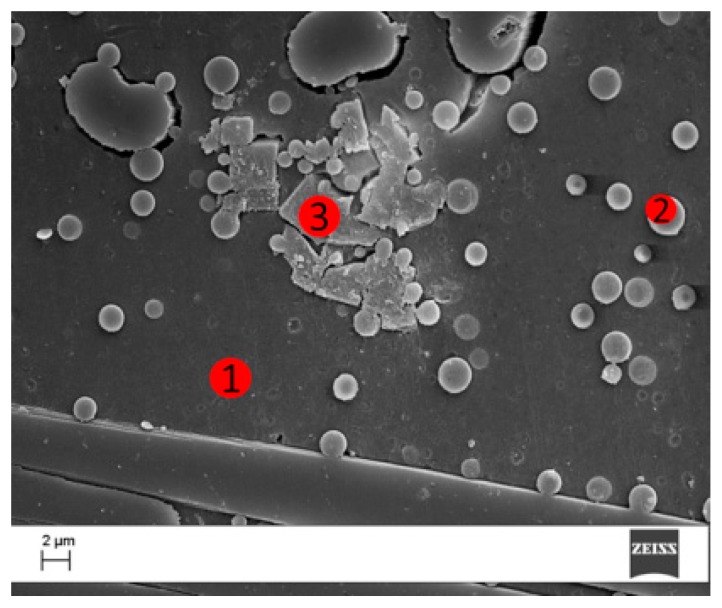
SEM images of the region analyzed by EDX for the laminate obtained with the coPES/1% POSS veil.

An EDX analysis has been performed in three of the interlaminar regions characterized, respectively, by uniform resin morphology (Figure 11, zone 1), coPES spherical particles (zone 2), and POSS squared crystallites (zone 3). Table 2 reports the related quantitative analysis. The data reported showed the presence of Si and Cl in zone 3, which is clearly characterized by the presence of POSS agglomerates. The EDX analysis of zone 1 and 2 did not show clearly the presence of Si and Cl, but due to the resolution of the EDX, this result cannot be used to assess the presence or absence of POSS at the nanoscale. In zone 2, above the micrometer sphere, the EDX analysis revealed the presence of S, which is coming from the coPES polymer. The presence of a rich S area confirms that the spherical particles are coPES rich.

**Figure 12:**
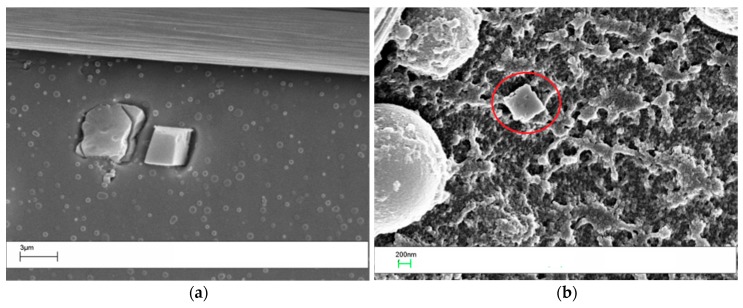
SEM of the laminate with the coPES/5% POSS veil dissolved in it: (**a**) low resolution (4000×, sx) and (**b**) high resolution (60,000×, dx).

**Figure 13:**
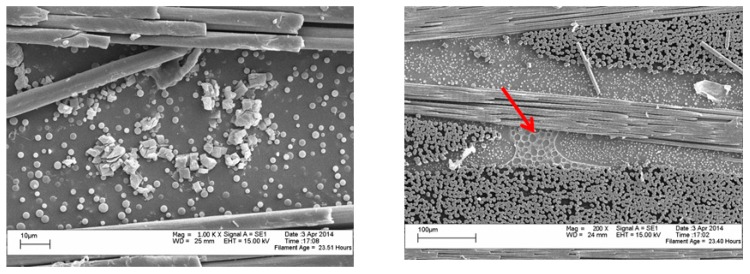
SEM of the laminate with the coPES/10% POSS veil dissolved in it: High resolution (1000×, sx) and low resolution (200×, dx).

The laminates prepared by adding 10 wt % of the veil coPES/5% POSS (Figure 12) and coPES/10% POSS (Figure 13) showed morphologies that deviated from the morphological behavior of the sample with the coPES veil only. The morphology for the coPES/5% POSS appeared particulate, with smaller coPES particles (<1 µm) and micro squared POSS crystallites (Figure 2a). High resolution SEM showed nanoscaled POSS crystallites in this case, too (Figure 12b).

**Figure 14:**
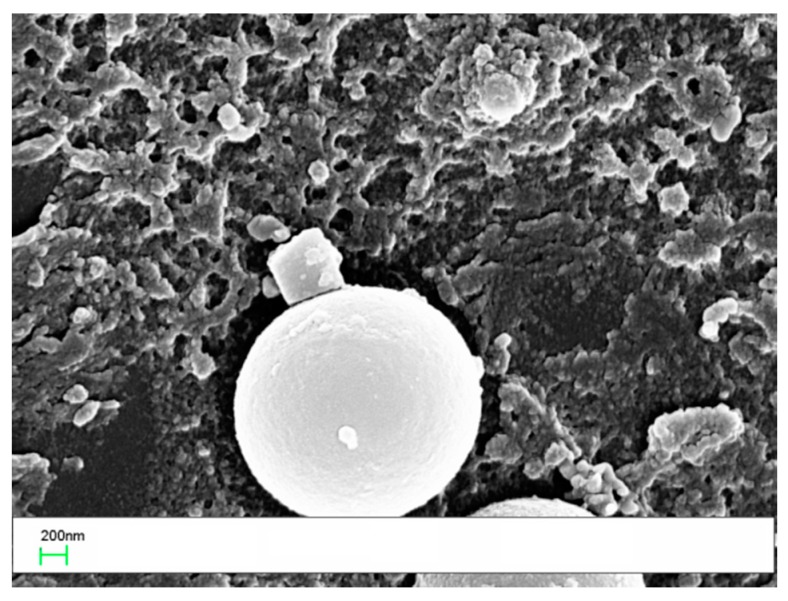
Higher resolution SEM of the laminate with the coPES/10% POSS veil dissolved in showing nanoscaled POSS (60,000×).

When the coPES/10% POSS veil was used, a dominant particulate morphology (Figure 13a) and some regions with salami morphology (Figure 13b) were observed. When high resolution SEM was performed on this sample, nanoscaled POSS were found again (Figure 14).

The analysis of all the laminates with coPES/POSS veils revealed (i.e., Figure 2) the presence in the interlaminar region of microscaled POSS agglomerates, confirming that POSS can be released in the interlaminar region upon the dissolution of the nanofibers by the curing epoxy resin. The same mechanism is thought to occur for the nanoscaled POSS, which are also seen in the electrospun veils.

The morphological differences observed in the interlaminar region for the samples with coPES/POSS veils, if coupled with the delayed dissolution times (i.e., Figure 7), revealed that POSS influenced the dissolution/phase separation mechanism. In particular, the interaction of POSS molecules with coPES chains can hinder its diffusion, resulting in slower dissolution and phase separation kinetics, which results in a smaller or non-uniform particulate morphology. In addition to that, the presence of beads rich in microscaled POSS could have impacted the homogenous dispersion of the coPES, resulting in an area with phase-inverted or salami morphologies.

### 3.3. DMA Properties of Composites

**Figure 15:**
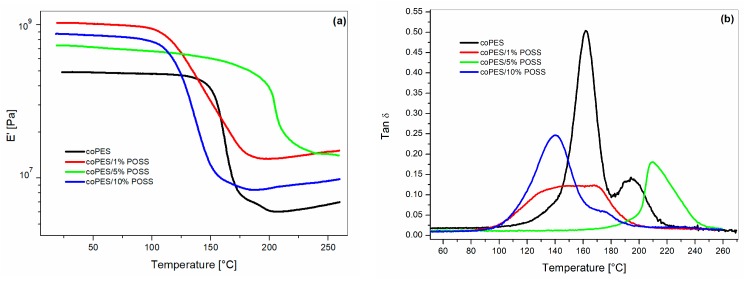
DMA analysis of the cured laminates: (**a**) storage modulus (*E*’) vs. *T*; (**b**) tan δ vs. *T*.

The viscoelastic properties of the POSS epoxies were measured by DMA (Figure 15). DMA is sensitive to molecular-level heterogeneities, and can give insight into the epoxy/coPES/POSS network architecture. The sample containing the coPES veil showed (Figure 15b) the presence of two distinct tan δ peaks at 161 and 193 °C, corresponding to the α-relaxations of the epoxy-rich and the coPES-rich phases, respectively. The laminates cured with coPES/POSS veils showed more complex behavior. In the glassy and rubbery regions, the samples with coPES/POSS veils showed a higher storage modulus (*E*’). The increase of the storage modulus was the result of the reinforcing effect of the POSS cages. As explained by Lee and Lichtenhan [15], the presence of POSS cages hinders the motions of molecular chains and network junctions. In addition to that, it can be noted that the microscopic POSS agglomerates, due to the stiff inorganic nature of POSS, could also contribute to the increase of the storage modulus. Similar results were also reported by Zhang et al. [33].

The tan δ curves showed a different behavior compared to the sample with the unfilled coPES veils. The addition of POSS resulted in the lowering of the amplitude of the tan δ peak for all POSS percentages. In addition to that, two tan δ were not evident in those samples. The sample with the veil coPES/1% POSS showed a broad single peak spanning from 100 to 200 °C. The sample with the veil coPES/5% POSS showed a sharper single peak centered at 210 °C, while when the POSS content increased to 10%, a main peak at 140 °C appeared with a shoulder at about 174 °C. In accordance with the findings of the morphological analyses, the DMA results confirmed that the addition of POSS interacted with the dissolution/phase separation behavior of the coPES nanofibers.

The composites used in this study were highly complex, due to the simultaneous presence of the coPES dissolution and phase separation phenomena. The formulation with a higher percentage of POSS segregates (i.e., CoPES/10% POSS, Figure 4) showed one tan δ peak and a shoulder. The particulate morphology for this system was similar to that of the unfilled coPES sample. Therefore, we can conclude that at high POSS percentages, the presence of more POSS segregates leads to a smaller influence on the dissolution/phase separation behavior, but, still, the presence of POSS dispersed in the epoxy and coPES phase affected the resultant viscoelastic behavior. In addition to that, the tan δ peak height was higher for the laminate with the coPES/10% POSS veils than for those with veils modified by 1% and 5% of POSS. This finding confirmed the minor presence of POSS nanocages for the sample with a higher number of segregates. The laminates obtained with veils containing 1% and 5% of POSS showed higher reductions for the tan δ peak, and its shape turned to be a single peak.

For the sample with coPES/1% POSS, the phase separation process led to a complex particulate morphology coupled with phase-inverted regions. As shown previously [34,35], the viscoelastic behavior of coPES/Epoxy blends is sensitive to the blend’s morphology. Therefore, the tan δ trace is the combined result of the peculiar morphology and the dispersion of nanogaged POSS.

The sample with coPES/5% POSS had even smaller and uniform coPES particles than for the other samples, which can justify the presence of a single peak in the tan δ trace. The shift to a higher temperature of the tan δ peak could be the result of a higher amount of CoPES dissolved in the epoxy-rich sample and of the presence of a higher amount of POSS nanocage reinforcement.

### 3.4. Thermal Stability

**Figure 16:**
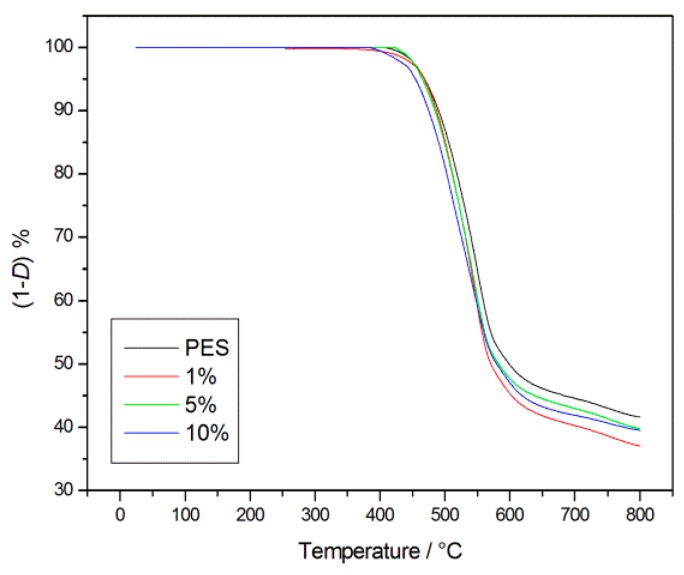
Thermogravimetric Analysis (TGA) of the coPES electrospun veils.

The thermal behaviour of the composites was investigated, together with that of the pristine PES, which was also studied for comparison. The aim was to determine if, and if so, and by how much the resistance to the thermal degradation is affected by the introduction into the polymer matrices of different percentages of nanoscale POSS molecules. Both the PES and composites degraded in a well-defined single stage, thus leading us to select the initial decomposition temperature (*T*_i_) to evaluate the resistance to thermal degradation (Figure 16). The *T*_i_ values are graphically determined through degradation TG curves as the intersection between the starting mass line and the maximum gradient tangent to the curve [26]. The TG curves, reported in Figure 16, clearly show that the *T*_i_ values obtained for all coPES/POSS (478, 472, and 464 °C for composites at 1%, 5%, and 10% of POSS respectively) were slightly lower than that of PES (480 °C), thus indicating that the dispersion of nanoparticles in the polymer matrix does not compromise significantly the thermal stability of the obtained composites.

## 4. Conclusions

The purpose of the present paper was to evaluate the feasibility of soluble thermoplastic veils as a means to disperse, in the interlaminar region of composite, POSS fillers without adding them to the neat resin.

The morphological analysis carried out on the electrospun veils demonstrated that electrospinning is an efficient technique to obtain thermoplastic veils with a high content of POSS dispersed both at the nano and microscale.

The analysis on the cured laminates confirmed the full dissolution of the coPES nanofibers within the processing ranges used, and, of foremost importance, proved that nanofillers can be effectively dispersed by using the soluble thermoplastic veils. The addition of POSS showed to have a significant effect on the final interlaminar morphology, and, in turn, on the viscoelastic properties of the laminates. The veils filled with 5% of POSS showed the best improvement in terms of glass transition temperature because of the synergetic effect of morphology control and POSS dispersion level.

The results achieved in this paper could be further expanded by filling the coPES veils with different types of nanofillers, such as carbon nanotubes or nanoclay. The use of filled thermoplastic veils is a promising approach to develop laminates with multiple layers of different types of nanofillers in a single processing step.

## Figures and Tables

**Table 1:** EDX analysis of PES–POSS fibres (atomic percentage calculated by selecting seven areas per sample and three points per area in each sample).

POSS wt %	C	O	S	Si	Au	Cl
0	80	11.5	6	-	2.5	
1	80.3	11.9	6.1	0.1	1.6	
5	78.2	10.4	8.5	0.3	2.6	
10	78.4	10.5	7.6	0.4	2.6	0.5

**Table 2:** EDX analysis for the zones of the interlaminar region (see Figure 10) analyzed by EDX for the laminate obtained with the coPES/1% POSS veil.

Zone	C	O	S	Si	Au	Cl
1	88	10	-	-	2	-
2	86	10	2	-	2	-
3	67	19	-	8.2	5.1	0.7
